# Myasthenia-like paraneoplastic syndrome with multiple cranial nerve tumor infiltration: A case report and literature review

**DOI:** 10.1097/MD.0000000000033774

**Published:** 2023-05-17

**Authors:** Chunbei Wen, Jie Yang, Changyou Xu, Dongsheng Wei, Lijun Luo

**Affiliations:** a Department of Neurology, The First Hospital of Wuhan, Wuhan, P. R. China; b The First Clinical Medical Institute, Hubei University of Traditional Chinese Medicine, Wuhan, P. R. China.

**Keywords:** adenocarcinoma of lung, case report, myasthenia gravis, paraneoplastic syndromes, peripheral nervous system neoplasms

## Abstract

Approximately 0.001% of patients with cancer have paraneoplastic nerve system syndrome, which can affect the central nervous system, neuromuscular junction, or peripheral nervous system. Although myasthenia gravis (MG) may exist as a thymic paraneoplastic syndrome (PNPS), its association with primary lung cancer remains unknown.

**Patient concerns:**

A 55-year-old female presented with slurred speech, weakness in chewing, sporadic difficulty in swallowing, and weakness in both lower limbs for half a year.

**Diagnoses:**

Based on cerebrospinal fluid and electromyography findings, we present the case of a female patient diagnosed with overlapping multicranial nerve tumor infiltration and MG-like neurological PNPS secondary to lung adenocarcinoma.

**Interventions:**

The patient received intrathecal injections of pemetrexed and neurotrophic (vitamin B) therapy before ceasing chemoradiotherapy and chose cabozantinib on her own.

**Outcomes:**

Weakness of the proximal limbs, choking cough, and chewing problems did not improve significantly.

**Lessons:**

Although it is unclear why MG coexists with lung cancer, it is probable that MG is a paraneoplastic condition. Cerebrospinal fluid testing should be carried out along with electrophysiological, serological, and pharmacological procedures pertinent to the diagnosis of MG to thoroughly examine if people simultaneously experience MG-like PNPS and tumor growth. Starting immunotherapy and anticancer medication at the same time that tumor development and MG-like syndrome are discovered is crucial.

## 1. Introduction

Myasthenia gravis (MG) is a T cell-dependent, B cell-mediated, complement-involved autoimmune neuropathy characterized by acetylcholine receptor antibody at the neuromuscular junction or other components of the postsynaptic muscle endplate, leading to neuromuscular transmission disorders. MG manifests several typical features: Partial or systemic skeletal muscle weakness and easy fatigue; Aggravated symptoms following activities, and; Marked relief of symptoms after rest or application of cholinesterase inhibitors.^[1]^ MG can be diagnosed based on the typical characteristics of fluctuating myasthenia and either an electrophysiological or serum antibody detection test.^[2]^ A fast-acting acetylcholinesterase inhibitor administered intravenously can instantly reverse MG muscle weakness, but this type of diagnosis method is more subjective.

Approximately 10% to 15% of patients with MG have thymoma, and 40% of thymoma patients eventually develop MG.^[3]^ When combined with thymoma, MG exists as a paraneoplastic syndrome (PNPS) of thymoma. The association between MG and thymoma has been widely documented in the literature,^[4–6]^ its association with lung cancer has not been clearly described. Herein, we report a case of MG coexisting with lung adenocarcinoma.

## 2. Case presentation

A 55-year-old female patient was admitted to the hospital on May 11, 2022, due to “weakened chewing accompanied by dizziness and unsteady walking for nearly half a year”. Over the past 6 months, the patient gradually developed slurred speech, weakness in chewing, sporadic difficulty in swallowing, weakness in both lower limbs, and unsteady walking. Standing up from a sitting position made her dizziness more pronounced, which was likely caused by a shift in body position. The patient also had a dry nasal cavity that was prone to bleeding. In October 2011, the patient experienced right tinnitus, aural fullness, and hearing loss. She was diagnosed with “sudden deafness in the right ear” and received medical treatment. Subsequently, the hearing of the left ear gradually decreased, and no treatment was administered.

**Figure 1.**
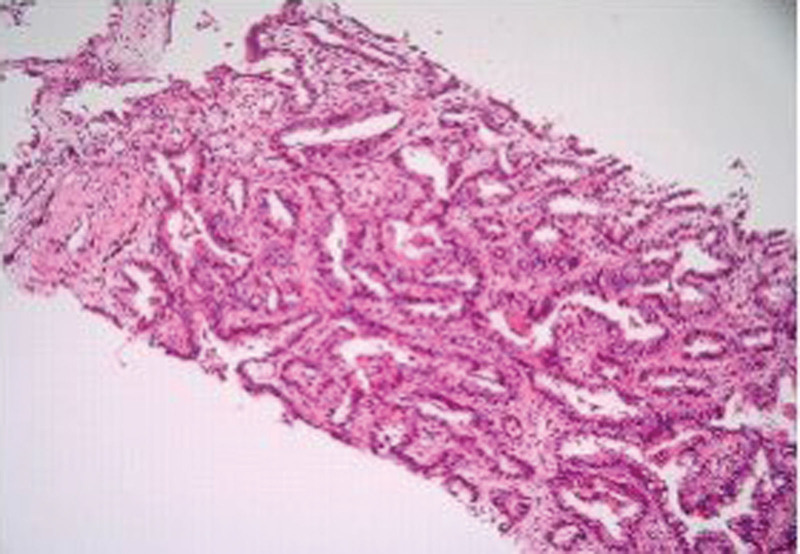
Right lung biopsy tissue (invasive lung adenocarcinoma) with predominantly acinar growth pattern.

On March 11, 2022, because of an “obvious decline in hearing and vision and weakness of limbs for nearly 3 months”, she returned to the hospital for medical investigation. Biological tests indicated that the cerebrospinal fluid (CSF) was colorless and transparent. The total number of white blood cells in the CSF was 23 × 10^6/L (0–5 × 10^6/L). The amount of protein was 0.63 g/L (0.15–0.45 g/L). Sugar and chloride levels were normal, and no tumor cells were observed. Today, the left ear is almost completely deaf, and the right ear functions to a very low degree, partly reserved, making communication almost impossible. She had been suffering from a lung tumor for nearly 4 years, and pathological examination revealed lung adenocarcinoma (Fig. 1). Next-generation sequencing revealed an Epidermal Growth Factor Receptor missense mutation (NM_005228.3), c.2573T > G mutation (p. Leu 858Arg), and t790m. The patient was treated with osimertinib 160 mg qd for tumor-targeted therapy. No surgery was performed and there was no other medical history.

Vital signs were stable with postural hypotension. The patient had slurred speech, dysarthria, and decreased visual acuity. She could normally frown and close her eyes but closed her lips loosely without an obvious decrease in facial pain sensation. The patient also had weakness in her cheeks and chewing problems. She was completely deaf on her left side and partially deaf on her right side. The patient’s gag reflex was sluggish. Her shoulder muscles were weak, and she could not extend her tongue past her lips, which was accompanied by atrophy and tremors. Limb proximal muscular strength was grade 4, and grade 5 distal muscular strength was present. The tendon reflexes of both upper and right lower extremities were +++, and her left lower extremity tendon reflexes were ++++. Both upper extremities passed the finger-nose test, and the heel-knee-shin test failed for both lower extremities. The pathological signs of the left lower extremity were suspiciously positive, Lasegue sign was (−), and meningeal irritation sign was (+).

**Figure 2.**
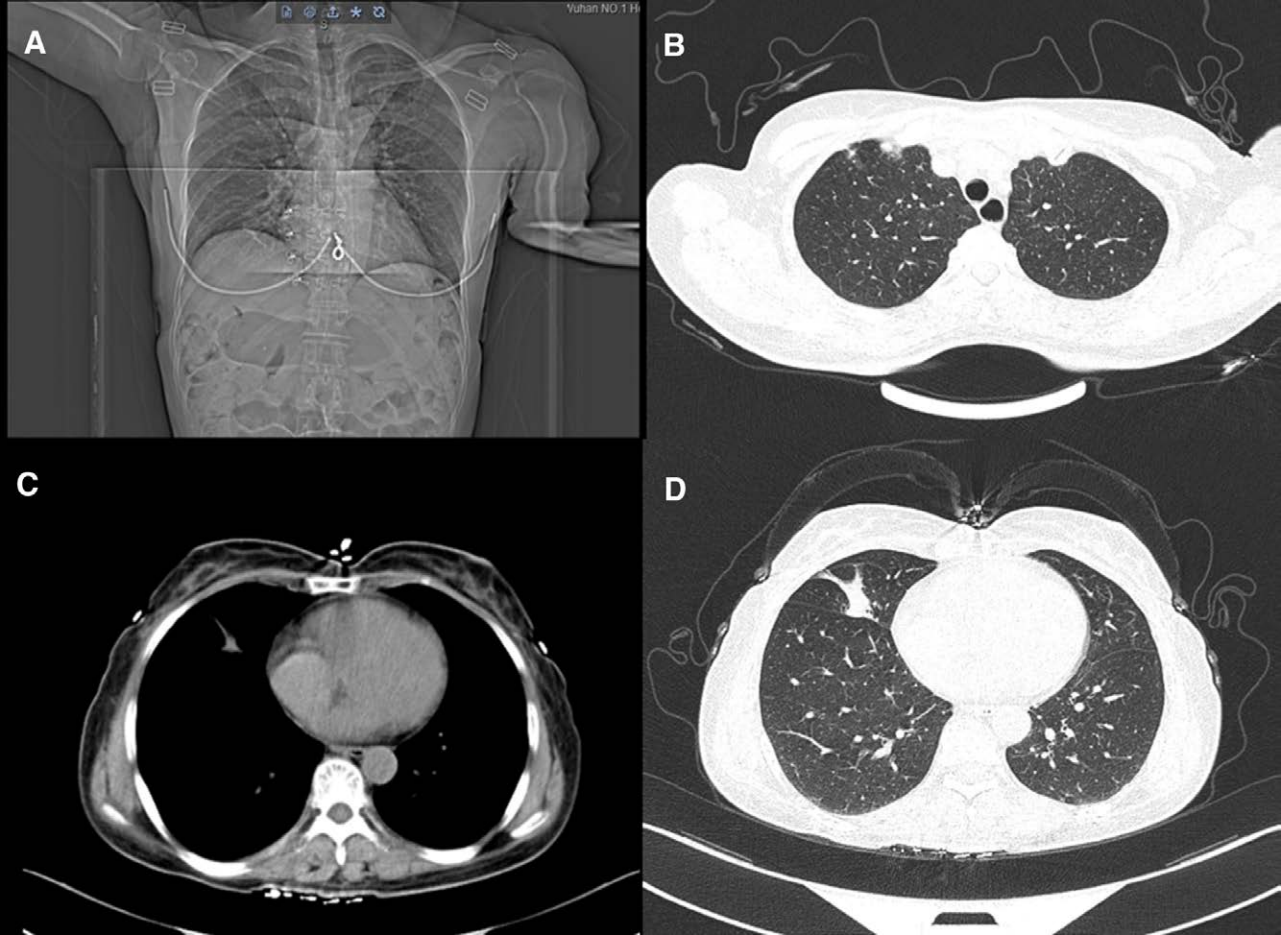
Lung CT: (A and B) Partial solid nodules in the upper lobes of both lungs, (C and D) thin-walled cavity in the right lower lobe. CT = computerized tomography.

**Figure 3.**
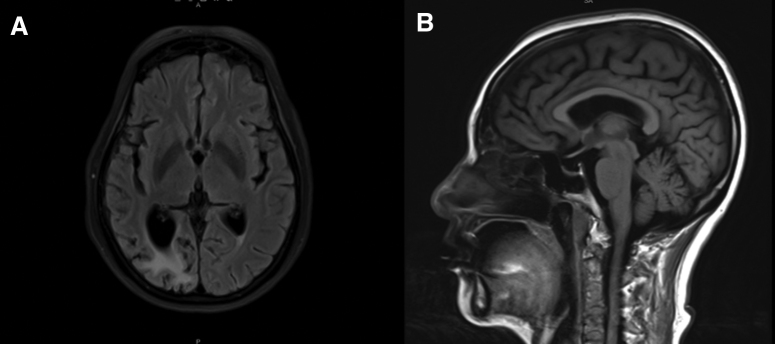
(A) Patchy long T2 and T2 Flair low signal shadows in the right occipital lobe, patchy edema around the posterior horn of the right lateral ventricle, no enhancement, (B) no cerebellar atrophy in the sagittal plane.

Computed tomography of the lungs revealed thin-walled cavities in the lower lobe of the right lung and scattered solid nodules over the entire lung (Fig. 2). Magnetic resonance imaging scans of the brain, nasopharynx, cervical spine, and thoracic spinal cord revealed several lacunar cerebral infarctions, but no evident mass in the nasopharynx. There was an aberrant signal shadow in the right occipital lobe but no augmentation (Fig. 3). The results of the neostigmine test were negative. The following MG-related antibodies were absent: anti-acetylcholine receptor antibody, anti-muscle-specific tyrosine kinase autoantibody, anti-titin autoantibody, and anti-low-density lipoprotein receptor autoantibody. Anti-Hu, anti-Yo, anti-SOX1, and anti-voltage-gated calcium channel autoantibodies were negative for paraneoplastic antibodies.

**Figure 4.**
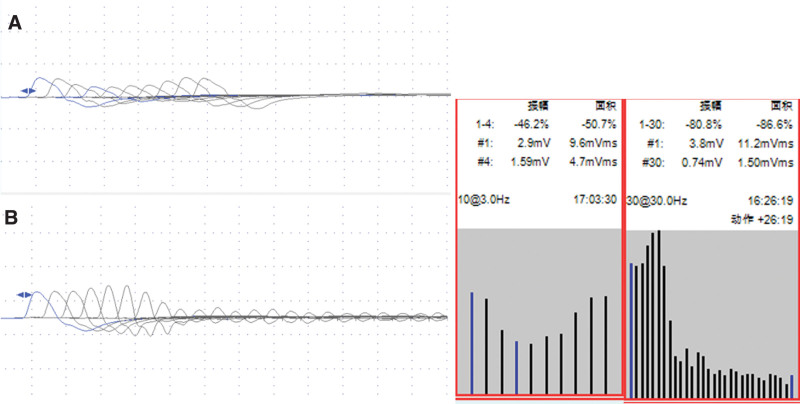
(A) The amplitude of the left abductor pollicis brevis muscle decreased by 46.2% after repeated stimulation at low-frequency (3Hz), (B) the amplitude decreased by 80.8% after repeated stimulation at high-frequency (30Hz).

**Figure 5.**
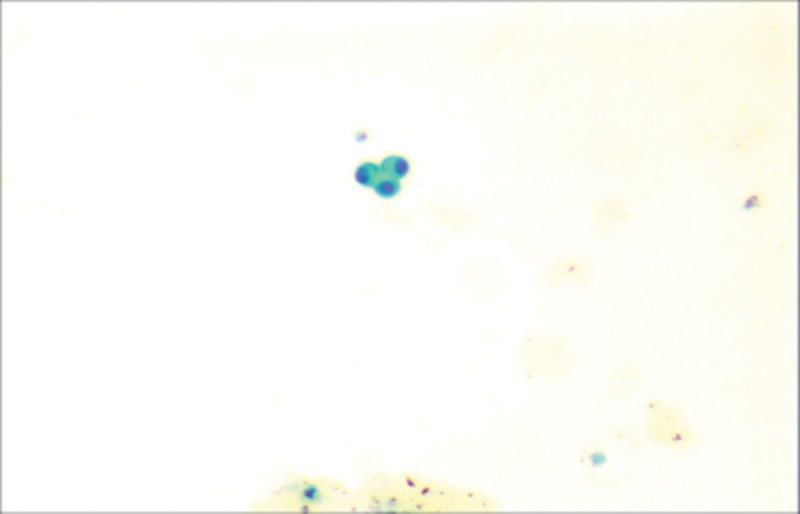
A cerebrospinal fluid smear shows a small number of monocytes, lymphocytes, and scattered epithelioid cells with mild atypia, tending to be poorly differentiated adenocarcinoma.

Electromyography (EMG) revealed that some of the muscles examined had compound muscle action potential (CMAP) amplitudes that were attenuated by low-frequency stimulation (3 Hz) (the left abductor pollicis brevis muscle was attenuated by 46.2%, the right orbicularis oculi muscle was attenuated by 11.6%, and the right frontalis muscle was attenuated by 11.4%). High-frequency stimulation (30 Hz) did not significantly enhance the CMAP amplitude of the tested muscle (left abductor pollicis brevis) (Fig. 4). Cerebrospinal fluid examination was repeated, and based on the pathological findings, scattered, mildly atypical epithelioid cells and poorly differentiated adenocarcinomas were considered (Fig. 5).

**Figure 6.**
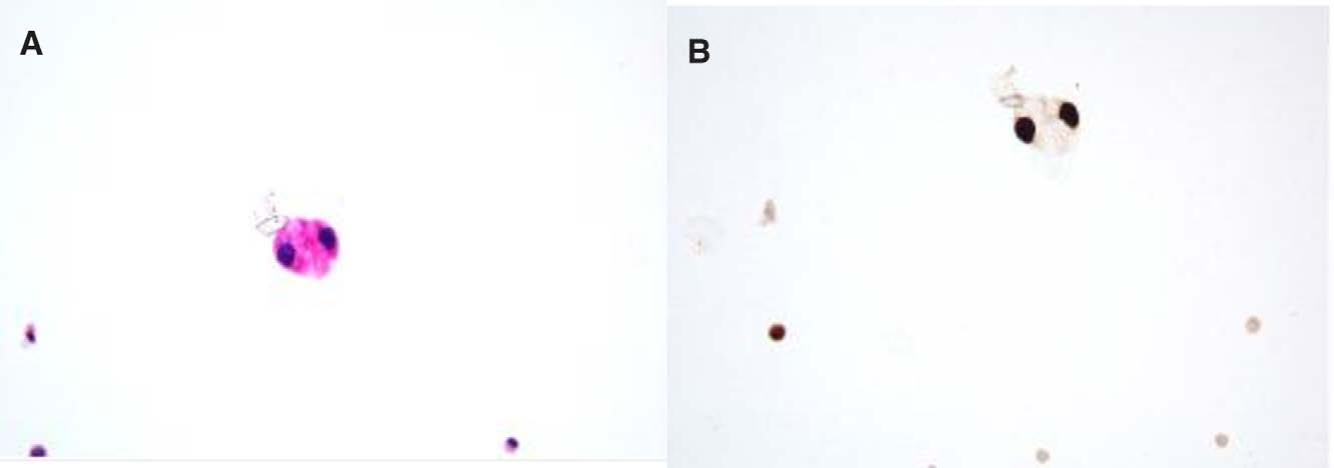
Cancer cells were again found in the cerebrospinal fluid, consistent with the lung adenocarcinoma phenotype.

The diagnoses were made as follows: Lung cancer with meningeal metastasis, multiple cranial nerve damage; Nervous system PNPS: secondary MG-like syndrome; Cerebellar and brainstem inflammation cannot be ruled out. Treatment should include supportive symptomatic care and immunotherapy in addition to treating the main tumor.^[7]^ She was treated with neurotrophic (vitamin B group) therapy and advised to undergo hormone therapy, but the patient declined. After discharge, she received intrathecal chemotherapy with Pemetrexed in Xiehe, and tumor cells were again found in the CSF (Fig. 6). Subsequently, the patient stopped chemoradiotherapy and switched to cabozantinib on her own.

One month later, family members complained that although the patient’s mental condition appeared to have marginally improved during chemotherapy, the patient’s proximal limb weakness, choking cough, and chewing difficulties persisted.

Informed consent was obtained from the patient for publication of the findings of this case report.

## 3. Discussion

PNPS can affect various organ systems, most notably the endocrine, neurological, dermatological, rheumatological, and blood systems. They are brought on by hormones, peptides, or cytokines secreted by tumors, or by immune cross-reactivity between malignant and normal tissues. Tumor neural antibodies can often be detected in the serum, but 30% of paraneoplastic nerve system syndrome (PNS) patients fail to detect related antibodies.^[7]^ Common PNS, such as central nervous system syndrome (e.g., limbic encephalitis and paraneoplastic subacute cerebellar degeneration), neuromuscular junctions (e.g., Lambert-Eaton myasthenic syndrome [LEMS]), peripheral nervous system (e.g., autonomic neuropathy and subacute sensory neuropathy), and retinopathy are often prone to occur.^[8]^

A diagnosis of common PNS, such as LEMS and subacute cerebellar degeneration, was considered in this patient due to decreased muscle strength and ataxia, but this was not confirmed by related examinations. Re-frequency EMG revealed that CMAP waves were attenuated after low-frequency and high-frequency stimulation, which is consistent with the typical electrophysiological characteristics of MG.^[9]^ Simultaneously, tumor cells were found in the CSF. Experts from multiple disciplines, including neurology, EMG, and oncology, have discussed this case. They ultimately agreed that cranial nerve injury caused by tumor infiltration could simultaneously involve the motor and sensory nerves. Owing to the influence of gravity, the latter group of cranial nerves is easily involved, and nerve injury is often asymmetric. There were pure sensory nerves II and VIII in this patient, mixed nerves V for the maxillary and mandibular branches, and X for cranial nerve injury. Multiple cranial nerve tumor infiltrations cannot be ruled out because MG syndrome is unable to fully explain the patient’s symptoms. In addition, the patient had instability in walking, Romberg sign was unstable with both eyes open and closed, and tendon reflexes were hyperactive. Although no abnormality was found on head Magnetic resonance imaging, brainstem and cerebellar inflammation due to tumor infiltration could not be excluded.

Taken together, our analysis and treatment provide new insights into our understanding of tumor-related MG-like neurological syndromes and the infiltration of cancer cells with neurological damage. In addition, brainstem and cerebellar inflammation caused by tumor infiltration cannot be completely excluded.

In small-cell lung cancer, PNPS is most frequently linked to lung cancer, with LEMS being the most prevalent PNS in the neuromuscular junction. When limiting the search time to 1990 to 2022, the search terms to “Lung Neoplasms” or “Adenocarcinoma of Lung” AND “myasthenia gravis” in Pubmed, most of the published cases are reports of other lung tumors complicated with MG,^[10–21]^ but only 3 cases of lung adenocarcinoma^[22–24]^ (Table 1). A thorough assessment of the literature led us to conclude that the causes of the coexistence of the 2 components have steadily become better understood over time, moving from an accidental phenomenon to an immunological problem to a paraneoplastic manifestation.

**Table 1:** Reported cases of lung cancer with myasthenia gravis.

Age/sex	Histology of cancer	Clinical manifestations	MG type	Anti-nAchR antibody	Electromyography	Neostigmine or edrophonium test	CSF	Thymoma	Order of occurrence	Treatment		Results		Author's think	Year	Reference
										For MG	For LC	For MG	For LC			
55 F	Ad	Muscle fatigue after chewing, slurred speech, and unsteady walking, with proximal limb weakness	General	−	+	−	+	−	MG developed 4 yr after lung cancer	−	ChT	Unchanged	NA	Need to start two treatments at the same time	2022	This case
71 M	SCLC	Ptosis, dysphagia, muscle fatigue after chewing	General	+	ND	+	ND	−	Synchronous	Py, steroid, tacrolimus, IVIG	ChT + RT	IMPRV	Stability	Need to start two treatments at the same time	2018	^[10]^
60 M	SCLC	Ptosis, poor speech, weakness of limbs	General	+	+	+	−	−	Lung cancer developed 11 mo after MG	Py, steroid	ChT	IMPRV	Stability	Perhaps a paraneoplastic syndrome	2018	^[11]^
64 M	NEC	Ptosis, diplopia, weakness of limbs	General	−	+	+	−	−	Synchronous	Py, steroid	ChT + RT	Worsen	PR	Perhaps a paraneoplastic syndrome	2015	^[12]^
38 M	Ad	Hemoptysis, chest pain, weakness of limbs	General	ND	+	ND	ND	−	Synchronous	−	−	Death		Perhaps a paraneoplastic syndrome	2014	^[22]^
69 F	Ad	Ptosis, weakness of limbs, dysphagia	General	+	+	ND	ND	+	Synchronous	Th	SX	Completely improve	Stability	Not sure if relevant	2012	^[23]^
56 M	SCLC	Ptosis, dysphonia, weakness of limbs	General	−, but Anti-Musk+	−	−	ND	−	Lung cancer developed 8 mo after MG	Py, steroid	ChT	Worsen	Death	Perhaps a paraneoplastic syndrome	2012	^[13]^
62 M	NSCLC	Ptosis, diplopia, weakness of limbs	General	+	+	ND	ND	−	Synchronous	ND	ND	ND	ND	Perhaps a paraneoplastic syndrome	2011	^[14]^
65 M	SCLC	Ptosis, weakness of limbs, polypnea	General	+	+	+	−	−	Synchronous	Py, steroid, PE	ChT	IMPRV	Death	ND	2011	^[15]^
55 M	NSCLC	Ptosis, axial muscle weakness	General	+	−	ND	ND	−	MG developed 2 years after surgery of lung cancer	Py, steroid, PE	ChT + RT + SX	IMPRV	ND	May be related to some kind of autoimmunity	2010	^[16]^
77 M	Sq	Bulbar palsy	General	+	+	ND	ND	−	Lung cancer developed 6 yr after MG	Py, steroid, Th	SX	IMPRV	ND	May be related to some kind of immune disorder	2006	^[17]^
46 M	La, Ad	Ptosis, diplopia	Ocular	+	ND	+	ND	−	Synchronous	Steroid, AZA, Th	RT + SX	Death		Perhaps a paraneoplastic syndrome	2000	^[24]^
56 M	AC	Ptosis, rhinolalia, facial weakness, weakness of limbs	General	+	+	ND	ND	−	Synchronous	Steroid, AZA, Th	SX	IMPRV	Survive	Happenchance	1999	^[18]^
44 F	Carcinoid	Diplopia, dysarthria, dysphagia, diarrhea	General	ND	+	ND	−	−	Synchronous	Py, loperamide, Th	SX	ND	ND	Happenchance	1997	^[19]^
56 M	SCLC	Ptosis, weakness of limbs, polypnea	General	−	+	+	ND	−	Lung cancer developed 18 mo after MG	Anticholinesterase inhibitors	ChT	IMPRV	Disappear	Cause unknown	1995	^[20]^
49 M	SCLC	Ptosis, progressive generalized muscle weakness, dyspnea	General	−	+	+	ND	ND	Synchronous	Edrophooium chloride	ND	IMPRV	ND	May be related to some kind of immune disorder	1994	^[21]^

AC = Atypical carcinoid, Ad = adenocarcinoma, anti-Musk = anti-muscle-specific tyrosine kinase autoantibody, AZA = azathioprine, CSF = cerebrospinal fluid, ChT = chemotherapy, F = female, IVIG = intravenous immunogloblin, IMPRV = improvement, La = large cell carcinoma, LC = lung cancer, M = male, MG = myasthenia gravis, NA = not available, ND = not described, NEC = poorly differentiated neuroendocrine carcinoma, NSCLC = non-small-cell lung cancer, Py = pyridostigmine, PE = plasma exchange, PR = partial response, RT = radiotherapy, Sq = squamous-cell carcinoma, SCLC = small-cell lung cancer, SX = surgery, Th = thymectomy.

In the case described by Wang et al,^[25]^ a patient was admitted to the hospital with ptosis and diplopia as the main complaints. Physical examination revealed lung cancer. Neostigmine text, MG, and paraneoplastic antibodies were all negative. EMG was not performed considering LEMS based on the symptoms alone. This finding is similar to the clinical features of our patient. Mesolella et al^[26]^ also reported a patient with laryngeal neuroendocrine carcinoma who did not undergo EMG examination and did not have paraneoplastic or MG-related antibodies. LEMS was diagnosed based on typical symptoms of proximal muscle weakness and autonomic dysfunction. As a result, we assume that the lack of prior reports on lung cancer and MG may be attributed to physicians propensity to make diagnoses; hence, the likelihood of diagnostic bias cannot be completely ruled out. Even in cases where MG syndrome has been identified, many people do not undergo CSF testing, which makes it difficult for doctors to properly comprehend the patient’s current condition. Furthermore, even in individuals with obvious imaging results and clinical symptoms, the positive diagnosis rate of CSF cytopathology is typically <50%, necessitating repeated testing.^[27]^

Successful cancer treatment does not always result in improvements in neurological function, and the mainstay of PNS treatment is immune-modulating drugs.^[8]^ In patients with PNS, combination therapy targeting malignancy and immunomodulation may help improve functional status.^[28]^ After discharge, the patient only received chemotherapy and targeted therapy for tumor metastasis and lacked immunotherapy for MG, so her symptoms did not improve dramatically.

Thus, clinicians should initiate antitumor therapy and immunotherapy simultaneously when tumor progression and MG-like syndrome are detected, which may improve the prognosis of patients. Our clinical reports and investigations may provide new insights for the near future. This is because MG will become an item on the list of PNS associated with lung tumors, along with LEMS and paraneoplastic cerebellar degeneration.

## 4. Conclusion

We report a case of a Chinese female patient with lung adenocarcinoma who may be associated with MG, although the reason for their coexistence remains unknown. More fundamental studies on PNPS are needed to clarify the probable link between the 2. CSF testing is critical for determining tumor progression and requires several examinations.

## Acknowledgments

The authors thank the patient’s family members for their cooperation in providing the medical data necessary for this publication.

## Author contributions

**Investigation:** Chunbei Wen, Changyou Xu, Dongsheng Wei.

**Validation:** Changyou Xu, Dongsheng Wei.

**Writing – original draft:** Chunbei Wen.

**Writing – review & editing:** Jie Yang, Lijun Luo.

## References

[1] Gilhus NE. Myasthenia gravis. N Engl J Med. 2016;375:2570–81. 28029925 10.1056/NEJMra1602678

[2] Gilhus NE Tzartos S Evoli A. Myasthenia gravis. Nat Rev Dis Primers. 2019;5:30. 31048702 10.1038/s41572-019-0079-y

[3] Toothaker TB Rubin M. Paraneoplastic neurological syndromes: a review. Neurologist. 2009;15:21–33. 19131854 10.1097/NRL.0b013e3181870aa2

[4] Basse C Girard N. Thymic tumours and their special features. Eur Respir Rev. 2021;30:200394. 34670805 10.1183/16000617.0394-2020 PMC9488894

[5] Ke J Du X Cui J. LncRNA and mRNA expression associated with myasthenia gravis in patients with thymoma. Thorac Cancer. 2022;13:15–23. 34773374 10.1111/1759-7714.14201 PMC8720629

[6] Yasumizu Y Ohkura N Murata H. Myasthenia gravis-specific aberrant neuromuscular gene expression by medullary thymic epithelial cells in thymoma. Nat Commun. 2022;13:4230. 35869073 10.1038/s41467-022-31951-8 PMC9305039

[7] Pelosof LC Gerber DE. Paraneoplastic syndromes: an approach to diagnosis and treatment. Mayo Clin Proc. 2010;85:838–54. 20810794 10.4065/mcp.2010.0099 PMC2931619

[8] Shamji FM Beauchamp G Maziak DE. Paraneoplastic syndromes in lung cancers: manifestations of ectopic endocrinological syndromes and neurologic syndromes. Thorac Surg Clin. 2021;31:519–37. 34696864 10.1016/j.thorsurg.2021.06.001

[9] Katzberg HD Abraham A. Electrodiagnostic assessment of neuromuscular junction disorders. Neurol Clin. 2021;39:1051–70. 34602214 10.1016/j.ncl.2021.06.013

[10] Yamasaki M Funaishi K Saito N. Acetylcholine receptor antibody-positive myasthenia gravis associated with small-cell lung cancer: a case report. Medicine (Baltim). 2018;97:e0541. 10.1097/MD.0000000000010541 PMC5944533 29703032

[11] Jia R Chen J Ge R. Coexistence of myasthenia gravis and Lambert-Eaton myasthenic syndrome in a small cell lung cancer patient: a case report. Medicine (Baltim). 2018;97:e10976. 10.1097/MD.0000000000010976 PMC5999448 29879051

[12] Niimi K Nagata E Murata N. Lung cancer associated with seronegative myasthenia gravis. Intern Med. 2015;54:1381–4. 26027991 10.2169/internalmedicine.54.3363

[13] Basta I Nikolic A Losen M. MuSK myasthenia gravis and Lambert-Eaton myasthenic syndrome in the same patient. Clin Neurol Neurosurg. 2012;114:795–7. 22421253 10.1016/j.clineuro.2011.12.044

[14] Shaygannejad V Ghasemi M Rajaee Z. Myasthenia gravis as a presenting feature in a patient with lung cancer: a case report. J Res Med Sci. 2011;16:229–32. 22091237 PMC3214309

[15] Ohira M Jeong D Oh SJ. Seropositive myasthenia gravis associated with small-cell lung carcinoma. J Clin Neurol. 2011;7:43–6. 21519527 10.3988/jcn.2011.7.1.43 PMC3079160

[16] Peltier AC Black BK Raj SR. Coexistent autoimmune autonomic ganglionopathy and myasthenia gravis associated with non-small-cell lung cancer. Muscle Nerve. 2010;41:416–9. 19882640 10.1002/mus.21528 PMC3925506

[17] Sakamaki Y Yoon HE Oda N. Non-small-cell lung cancer associated with non-thymomatous myasthenia gravis. Jpn J Thorac Cardiovasc Surg. 2006;54:207–11. 16764310 10.1007/BF02670314

[18] Burns TM Juel VC Sanders DB. Neuroendocrine lung tumors and disorders of the neuromuscular junction. Neurology. 1999;52:1490–1. 10227641 10.1212/wnl.52.7.1490

[19] Arruda WO Rosa JF. A 44-year-old woman with diarrhoea and double vision. Lancet. 1997;349:1882. 9217761 10.1016/S0140-6736(97)04392-4

[20] Miyoshi R Yamaji Y Shima S. [A case of small cell lung cancer that developed during therapy for myasthenia gravis]. Nihon Kyobu Shikkan Gakkai Zasshi. 1995;33:456–62. 7791277

[21] Fujita J Yamadori I Yamaji Y. Myasthenia gravis associated with small-cell carcinoma of the lung. Chest. 1994;105:624–5. 8306783 10.1378/chest.105.2.624

[22] Eivaz-Mohammadi S Gonzalez-Ibarra F Hekmatjou H. Myasthenia gravis-like syndrome presenting as a component of the paraneoplastic syndrome of lung adenocarcinoma in a nonsmoker. Case Rep Oncol Med. 2014;2014:703828. 25136468 10.1155/2014/703828 PMC4129963

[23] Takizawa M Oda M Matsumoto I. Myasthenia gravis complicated with lung cancer and middle mediastinal thymoma. Asian Cardiovasc Thorac Ann. 2012;20:486–8. 22879565 10.1177/0218492312440264

[24] Leavitt JA. Myasthenia gravis with a paraneoplastic marker. J Neuroophthalmol. 2000;20:102–5. 10870923 10.1097/00041327-200020020-00008

[25] Wang A Zhang X Yi J. Successful treatment of advanced lung adenocarcinoma complicated with Lambert-Eaton myasthenic syndrome: a case report and literature review. Thorac Cancer. 2020;11:1334–8. 32154996 10.1111/1759-7714.13385 PMC7180587

[26] Mesolella M Allosso S Buono S. Neuroendocrine carcinoma of the larynx with Lambert-Eaton myasthenic syndrome: a rare case report and literature review. J Int Med Res. 2021;49:3000605211014784. 33983073 10.1177/03000605211014784 PMC8127768

[27] Gao N Xin T. [Advances in diagnosis and treatment of leptomeningeal metastasis of lung cancer]. Zhongguo Fei Ai Za Zhi. 2022;25:517–23. 35899451 10.3779/j.issn.1009-3419.2022.102.25 PMC9346159

[28] Cheng YC Chang A Hsu WC. Anti-SOX1 antibody-positive paraneoplastic syndrome presenting with subacute cerebellar degeneration and lambert-eaton myasthenic syndrome: a case report. Acta Neurol Taiwan. 2021;30:74–7. 34549392

